# Editorial: Covid-19 Mechanisms on Cardio-Vascular Dysfunction: From Membrane Receptors to Immune Response

**DOI:** 10.3389/fcvm.2021.686495

**Published:** 2021-04-27

**Authors:** Amarylis C. B. A. Wanschel, Ana I. S. Moretti, Mireille Ouimet

**Affiliations:** ^1^Interdisciplinary Stem Cell Institute, Miller School of Medicine, University of Miami, Miami, FL, United States; ^2^Instituto de Assistência Médica ao Servidor Público Estadual (IAMSPE), Post-Graduation in Health Sciences Program, São Paulo, Brazil; ^3^University of Ottawa Heart Institute, Ottawa, ON, Canada; ^4^Department of Biochemistry, Microbiology and Immunology, Faculty of Medicine, University of Ottawa, Ottawa, ON, Canada

**Keywords:** COVID-19, SARS-CoV-2, endothelium dysfunction, hypertension, obesity, diabetes, cardiovascular disease

In this special issue of Frontiers in Cardiovascular Medicine, we assembled a collection of hypothesis and theory (1), methods (1), opinion (1), original research articles (1) and reviews (4), within an over-arching theme of “Exploring the COVID-19 mechanisms from membrane receptors to immune response” that uniquely reaffirmed our mission to understand and treat COVID-19. A comprehensive retrospective first takes us through the basic science of pre-existing diseases associated with increased COVID-19 severity, uncovering possible underlying mechanisms potentiating COVID-19 progression.

Coronavirus disease 2019 (COVID-19) has reached a global outbreak, increasing to pandemic proportion, and pre-existing chronic conditions such as cardiovascular disease, hypertension, diabetes, and obesity are major contributors to its morbidity and mortality worldwide (Figure 1). During severe acute respiratory syndrome coronavirus 2 (SARS-CoV-2) infection, enveloped, positive single-stranded RNA virus is endocytosed by epithelial and endothelial cells through the angiotensin-converting enzyme 2 (ACE2) receptor, requiring virus spike-ACE2 interactions and transmembrane serine protease-2 (TMPRSS2) priming (Figure 1). Expression of the ACE2 entry receptor for SARS-CoV-2 in vascular cells is upregulated in individuals with comorbidities, accelerating the course of disease development. Furthermore, direct viral infection of cardiomyocytes is possible due to ACE2 expression in the heart (Figure 1). In contrast, with less functional ACE2, the pediatric population appears to be less vulnerable to COVID-19 infection than adults.

**Figure 1:**
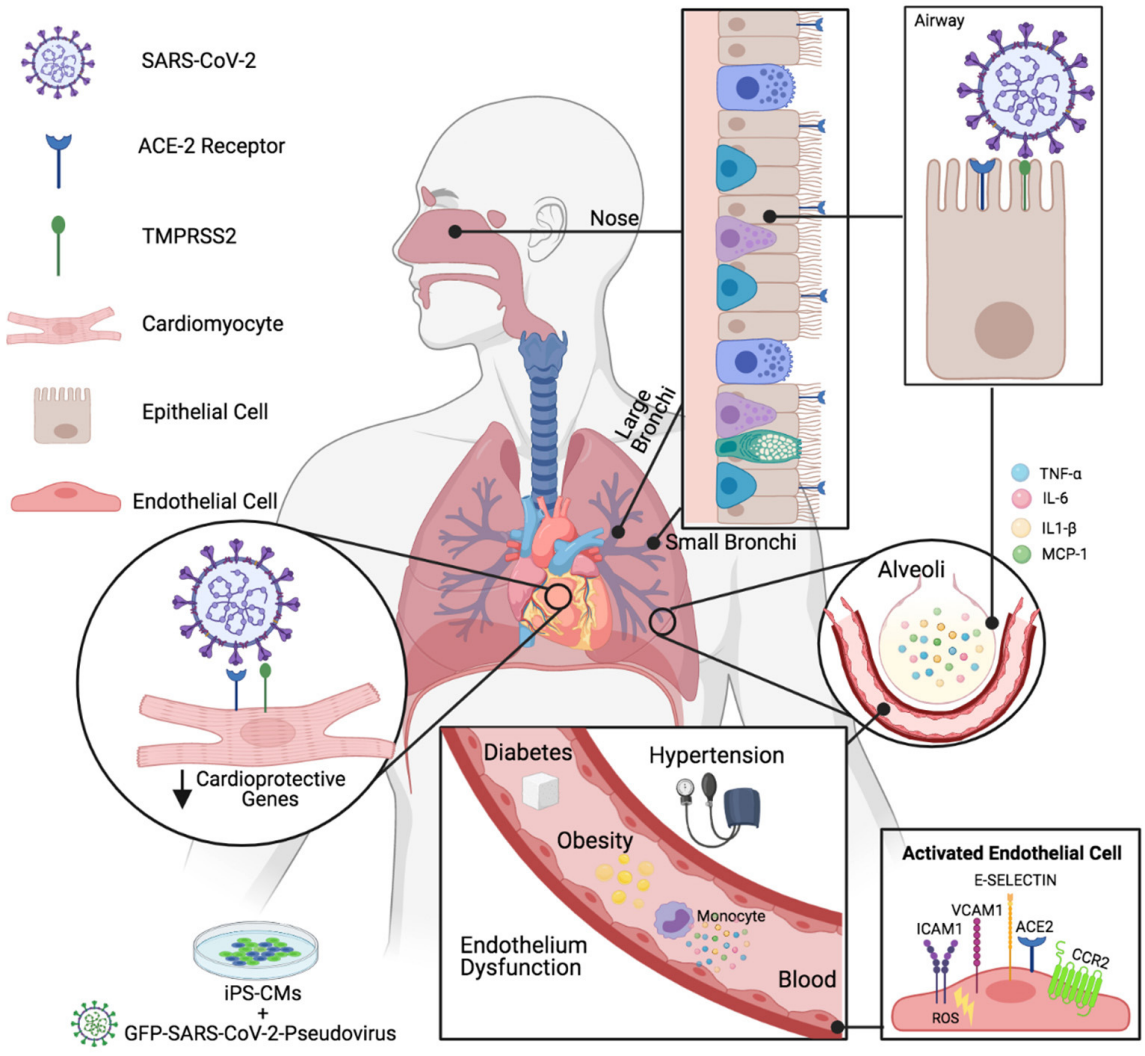
COVID-19 on Comorbidities (created with Biorender.com). Cardiovascular injuries contribute to disease severity during SARS-CoV-2 infection. ACE2, Angiotensin-converting enzyme 2; TMPRSS2, Transmembrane serine protease 2; iPS-CMs, Human inducible pluripotent stem cell-derived cardiomyocytes; GFP-SARS-CoV-2, Green fluorescent protein bearing SARS-CoV-2 pseudovirus; TNFα, Tumor necrosis fator alfa; (IL)-6, (IL)-1β, Interleukin; MCP-1, Monocyte chemoattractant protein 1; VCAM-1, Vascular cell adhesion molecule-1; ICAM-1, Intercellular adhesion molecule-1; CCR2, Chemokine receptor type 2; ROS, Reactive oxygen species.

The molecular basis, potential pathways and the role of endothelium in the pathogenesis of cardiac and vascular injuries in COVID-19 were discussed in a new study by Hachim et al. Notably, the common cardiomyocyte protective genes *SON, OGT*, and *RORA* were significantly downregulated in cardiovascular comorbidities (acute coronary syndrome, heart failure, venous thromboembolism) compared to controls and in the healthy endothelium of African Americans compared to Caucasians, suggesting that individuals who have a pre-existing genetic propensity for low expression of these genes may be more susceptible to cardiac damage during COVID-19. Recent studies have shown that cardiovascular comorbidity plays a role in cardiac injury in COVID-19 patients (Iacobazzi et al.). Furthermore, SARS-CoV-2-induced downregulation of another set of cardioprotective genes *NDUFA4L2, NDUFB7, MRPS11*, and *HIKESHI*, concomitant with upregulation *CHD9, MTF2, RORA, MYC*, and *ETS1* may promote cardiac injury upon SARS-CoV-2 infection (Hachim et al.) (Figure 1).

Vascular dysfunction is the common link between comorbidities and increased severity of COVID-19 infection; however, the factors that trigger endothelium injury differ in every common comorbidity in patients with COVID-19. During diabetes, hyperglycemia is associated with massive circulating and endothelial protein glycosylation, leading to elevated oxidative stress that damages blood vessel linings. The role of endothelial cells in SARS-CoV-2 viral infection was extensively reviewed by Hol Fosse et al., highlighting endothelial cell tropism, detection and response, along with the breakdown of vascular function during systemic hyperinflammatory response in COVID-19, as prominent features of human infection. An opinion article by Sur et al. shed light on the underlying mechanisms by which diabetes might augment the severity of COVID-19. Hyperglycemia may have a strong association with low intracellular pH and could potentiate the SARS-CoV-2 infection (Sur et al.). In addition, hyperglycemia promotes the synthesis of inflammatory cytokines, and diabetic patients with COVID-19 are more susceptible to immune cell hyperactivation, or a cytokine storm (Sur et al.).

Type 1 or type 2 diabetic patients treated with angiotensin converting enzyme inhibitors (ACEIs) or angiotensin-receptor blockers (ARBs) exhibit increased ACE2 receptor expression and could therefore be at a higher risk of SARS-CoV-2 infection and severity of COVID-19. However, changes in the expression of ACE2 in diabetes vary across different organs, and the impact of such changes on COVID-19 severity are still under investigation. Other meta-analyses suggested no significant association between the use of ACEIs or ARBs. Roberts et al. also highlight that hyperglycemia impairs host defense, and that blood glucose itself is a key mediator of severe symptoms in diabetic patients infected with SARS-CoV-2. The endothelium of diabetic patients is already compromised by hyperglycaemia-induced oxidative stress, driving endothelium activation, and increasing the expression of adhesion molecules, such as vascular cell adhesion molecule 1 (VCAM-1), intercellular adhesion molecule 1 (ICAM-1) and E-Selectin that orchestrate leukocyte recruitment (Figure 1).

Chronic and persistent immune dysregulation in type 1 or type 2 diabetes includes lower rates of phagocytosis by neutrophil and macrophages that is necessary for uptake of infectious antigen, in addition to suppressed chemokine responses and higher levels of pro-inflammatory mediators. Because lower respiratory tract infections occur when the immune response in the upper airways fails to contain the viral spread, the route of entry of SARS-CoV-2 in the lungs and the intimate proximity of the endothelium expressing ACE2 and alveolar epithelial cells together with resident macrophages compound COVID-19 severity in diabetic patients. Lung autopsies of deceased COVID-19 patients demonstrated that neutrophil extracellular traps (NETs), which are networks of extracellular fibers composed of DNA from neutrophils that bind pathogens, were likely to be involved in inflammation-associated lung damage, thrombosis, and fibrosis. To date, generation of NETs is a common feature in diabetes and infection, boosted by COVID-19. In diabetic patients, a potential primer for thrombosis in COVID-19 is increased circulating inflammatory cytokines, along with endothelial dysfunction as a crucial determinant of thrombotic potential through loss of anti-thrombotic nitric oxide (NO), prostaglandin 2 (PGI2), imbalance of fibrinolytic plasminogen activator inhibitor t-PA/PAI-1, and platelet hyperactivity, all of which are exacerbated by oxidative stress (Sur et al.).

The luminal surface of vascular endothelial cells is covered by a gel-like of interconnected proteins, the glycocalyx that plays a role in vascular permeability, adhesion of leucocytes and platelets, and modulation of inflammatory processes. Plasma-derived albumin is homogeneously distributed onto the endothelial glycocalyx layer (EGL). Interestingly, SARS-CoV-2 virions are transported by albumin, inhibiting free albumin binding to glycocalyx and disrupting normal fluid homeostasis of the microvasculature (Johnson et al.). Therefore, albumin therapy to replace SARS-CoV-2-bound albumin may improve vascular permeability and alleviate some of the COVID-19 symptoms leading to sepsis.

More studies are needed to evaluate the effect of SARS-CoV-2 and potential therapies on populations classified as risk groups. Capcha et al. have developed a protocol for *in vitro* studies of SARS-CoV-2 pseudovirus infection in renal and lung epithelial cells and in human induced pluripotent stem cell-derived cardiomyocytes (iPS-CMs) (Figure 1). Such models will prove useful to further evaluate the potential for therapeutic approaches to control SARS-CoV-2 infection, such as the recently described ultraviolet light that could modulate morbidity and mortality from COVID-19 (Gorman et al.) and cell-based therapy to attenuate cytokine storm (Iacobazzi et al.). Further understanding of the interactions of COVID-19 with the cardiovascular system will help the design of future therapeutic approaches to reduce morbidity in patients with underlying cardiovascular complications.

## Author Contributions

The authors equally contributed to the drafting revision of the editorial and approved it for publication.

## Conflict of Interest

The authors declare that the research was conducted in the absence of any commercial or financial relationships that could be construed as a potential conflict of interest.

